# Nanostructured Antimicrobials for Quality and Safety Improvement in Dairy Products

**DOI:** 10.3390/foods12132549

**Published:** 2023-06-29

**Authors:** Adriano Brandelli, Nathalie Almeida Lopes, Cristian Mauricio Barreto Pinilla

**Affiliations:** 1 Laboratory of Nanobiotechnology and Applied Microbiology, Department of Food Science, Federal University of Rio Grande do Sul, Porto Alegre 91501-970, Brazil; natilopes.nl@gmail.com (N.A.L.); cristianmaobarreto@gmail.com (C.M.B.P.); 2 Dairy Technology Center, Institute of Food Technology, Campinas 13083-015, Brazil

**Keywords:** dairy industry, nanostructures, natural antimicrobials, food safety

## Abstract

In the food sector, one of the most important economic activities is the dairy industry, which has been facing many challenges in order to meet the increasing demand by consumers for natural and minimally processed products with high quality. In this sense, the application of innovative and emerging technologies can be an interesting alternative, for example, the use of nanotechnology in packaging and as delivery systems. This technology has the potential to improve the quality and safety of dairy products, representing an interesting approach for delivering food preservatives and improving the mechanical, barrier and functional properties of packaging. Several applications and promising results of nanostructures for dairy product preservation can be found throughout this review, including the use of metallic and polymeric nanoparticles, lipid-based nanostructures, nanofibers, nanofilms and nanocoatings. In addition, some relevant examples of the direct application of nanostructured natural antimicrobials in milk and cheese are presented and discussed, as well as the use of milk agar as a model for a preliminary test. Despite their high cost and the difficulties for scale-up, interesting results of these technologies in dairy foods and packaging materials have promoted a growing interest of the dairy industry.

## 1. Introduction

The dairy industry has been undergoing a period of market expansion and consolidation during the last decades, which has provided several commercial opportunities and also diverse challenges related to safety, sustainability and innovation. Milk, the essential raw material of the dairy industry, contains relatively high amounts of fat, proteins and sugars and therefore is highly susceptible to deterioration [1]. Oxidative and hydrolytic reactions triggered by endogenous enzymes and enzymes from psychotropic bacteria have been associated with the development of unpleasant properties in milk and dairy products. In this regard, milk can be susceptible to contamination by a broad range of both pathogenic and spoilage microorganisms that cause significant losses to dairy products. The main concern is related to the fact that milk provides an excellent growth medium for major human pathogenic bacteria such as *Listeria monocytogenes*, *Staphylococcus aureus*, *Salmonella* spp. and toxigenic *Escherichia coli* strains [1,2].

Heat treatments have been long used to control microbial growth and some endogenous milk enzymes such as lipases, but there is increased concern about side reactions caused by ultrahigh temperature processing such as the formation of HMF (5-hydroxymethylfurfural) and acrylamide in dairy products [3]. Although pathogenic microorganisms are often destroyed by pasteurization, the recontamination of dairy products during post-pasteurization processing has been reported [2]. *L. monocytogenes* is a ubiquitous bacterium, showing a remarkable capability to survive under food-processing conditions such as low temperature and a relatively broad range of pH and salt concentrations [4]. *S. aureus* is a microorganism capable of producing thermostable enterotoxins, being an important etiological agent of bovine mastitis. Despite the low levels of staphylococcal enterotoxins found in dairy products, poor starter culture activity during fermentation and temperature abuse above 10 °C are factors involved in dairy-related outbreaks of staphylococcal intoxication [5,6]. *Salmonella* is found in the gastrointestinal tract of farmed and wild animals, and the most common causes of foodborne *Salmonella* infection are dairy products along with meat and eggs. In this context, inadequate pasteurization and post-process contamination have been associated as a cause of salmonellosis from milk and dairy products [7]. However, there is a low number of cases of severe disease caused by Shiga-toxin-producing *E. coli* in dairy products [8], probably due to an agreement of good hygienic practices at the farm and industry level. Thus, a combination of control methods and hurdle technology is important to avoid milk contamination. 

At the same time, consumers have an increased demand for natural and minimally processed food products, and in this sense, the application of innovative and emerging technologies can be an interesting alternative to deal with these issues, for example, the use of non-thermal processes like ohmic, high-pressure, ultrasound and membrane technology and nanotechnology [9]. 

In particular, nanotechnology has been considered a fascinating tool, showing great potential for the delivery and controlled release of natural preservatives in food [10]. This technology has the potential to improve the quality and safety of dairy products by carrying and protecting antimicrobial compounds against adverse environmental conditions that can appear during the production and storage of this type of food. Overall, nanotechnology represents an interesting approach to improving the quality, shelf life and safety of dairy products and the development of novel packaging materials with better mechanical, barrier and antimicrobial properties [11]. Therefore, there is an increasing interest of researchers and the dairy industry in exploring the potential of nanotechnology in the encapsulation and delivery of antimicrobial compounds, such as bacteriocins and essential oils, and the production of active packaging or coatings with antibacterial nanostructures. In this review, several applications and promising results of nanostructured antimicrobials in dairy products will be discussed. 

## 2. Natural Antimicrobials in Dairy Foods

The control of undesirable microorganisms can be achieved by chemical additives, but the use of natural compounds as antimicrobial agents is gaining increased interest. Antimicrobial peptides, essential oils and vegetable extracts have been tested as alternatives to control foodborne microorganisms [12]. 

As mentioned earlier, dairy products are highly susceptible to microbial deterioration during distribution and storage. Thermal treatments and fermentative processes are often used to control undesirable microorganisms in dairy foods, while combination with chemical preservatives like benzoic and sorbic acids and their salts, calcium lactate and calcium ascorbate is restricted [2,13]. A number of natural antimicrobial agents of plant, animal and microbial origin have been investigated as potential alternatives for microbiological control in the dairy industry, although the number of natural and chemical preservatives approved to protect dairy foods against microbial contamination is remarkably small [13]. 

Among antimicrobial agents of plant origin, several essential oils have been tested as natural agents for microbial control in cheeses, either by direct application in the food matrix or packaging incorporation. Essential oils mostly present a broad antimicrobial activity against bacteria, viruses and fungi, in addition to antiparasitic and insecticidal properties [14]. Essential oils obtained from rosemary, thyme, basil, oregano and some individual components like thymol and carvacrol can be effective to control most pathogenic bacteria that are prone to contaminate milk and dairy products [15,16]. Other alternatives are antimicrobials of microbial origin, such as nisin, pediocin, enterocins and the antifungal natamycin. The most used natural antimicrobial in the dairy industry is nisin, mainly as a preservative in heat-processed and low-pH foods. This substance is approved by the Food and Agriculture Organization/World Health Organization (FAO/WHO) and considered a food additive (E234) Generally Recognized As Safe (GRAS) by the FDA. Due to its activity against a wide variety of Gram-positive bacteria, nisin has received particular attention, showing effective inhibition of foodborne pathogens, such as *L. monocytogenes* and *S. aureus*, as well as spores of *Bacillus* and *Clostridium* [17]. Moreover, natamycin is widely used in the Asia Pacific and Europe, especially for cheese and sausages. Cheese is the main dairy product where natamycin has been employed to control fungal spoilage [18].

In addition, some natural antimicrobials of animal origin, including chitosan, lysozyme and lactoferrin, have also been used in dairy products such as cheese and milk [16,19]. Lysozyme is a naturally occurring antimicrobial in mammalian milk and can be utilized as a biopreservative in dairy products. Besides common strategies applied in cheese production, lysozyme can be used alone or combined with other approaches to improve cheese safety [20]. Lactoferrin exhibits bacteriostatic and bactericidal activity against several microorganisms because it binds iron, which is an element necessary for the growth of microorganisms like *L. monocytogenes*, *Salmonella* spp, *E. coli* and *Bacillus stearothermophilus*. In this way, lactoferrin may act as a natural antimicrobial for the biopreservation of dairy foods, such as yogurt, cheese, fermented milks and infant formulas [21].

The importance of natural preservatives has been recognized and has attracted a growing consumer interest. However, undesirable interactions with components of the food matrix and the degradation of antimicrobials by endogenous food enzymes, summed to possible negative effects on flavor characteristics, have been described for some natural compounds [22,23]. In this regard, nanotechnology arises as a promising alternative for the delivery of natural preservatives in foods. Nanostructured antimicrobials present some advantages in comparison with free antimicrobials, such as enhanced bioactivity, better chemical stability, controlled release and target delivery, resulting in improved properties to control spoilage and pathogenic microorganisms [24]. However, the selection of the appropriate system for nanoencapsulation may consider the interactions among the nanoparticulate material with the antimicrobial compounds and the food matrix. The research in this area has unraveled the potential of nanotechnology for the development of innovative vehicles for antimicrobial delivery and their application in several products, including dairy, meat, bread, juice and others, this being a current research topic in the field of food science and technology [10].

## 3. Nanostructures for Antimicrobial Delivery

In the last years, a great diversity of nanostructures has been developed as carriers and delivery systems of antimicrobial substances. Natural antimicrobial compounds, such as bacteriocins, essential oils, plant extracts and enzymes, can be incorporated into different nanostructures, according to their hydrophilic, lipophilic, anionic or cationic behavior. These physicochemical properties allow the selection of an appropriate nanostructure and method of preparation, ensuing materials with unique physical, chemical and biological features [25]. Properties such as the large surface–volume ratio, improved apparent solubility and low toxicity make the engineered nano-sized structures an attractive technology in agro-food sectors and several other industries [26].

**Figure 1:**
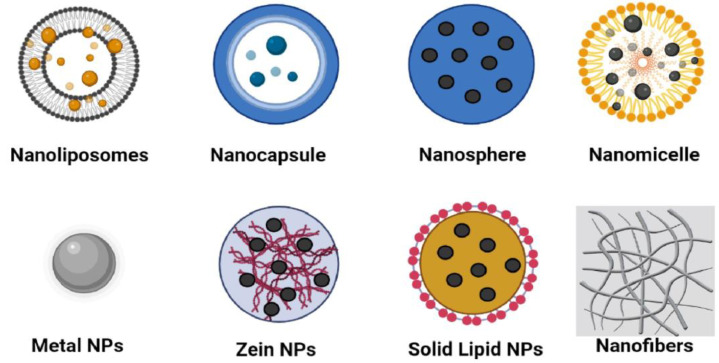
Schematic representation of different nanostructures available as nanocarriers for delivery of natural antimicrobials. Antimicrobials can be encapsulated into nanoliposomes, nanocapsules, nanospheres, nanomicelles, solid lipid nanoparticles and protein-based nanoparticles (like zein) or adsorbed onto surface of metal nanoparticles. Some metallic nanoparticles show antimicrobial activities by themselves. Antimicrobials can be encapsulated or adsorbed onto polymeric nanofibers, forming thin films for packaging purposes. Original image prepared by the authors.

A schematic representation of some nanostructures studied in the field of food science is presented in Figure 1. The suitability of a nanostructured system is related to the chemical properties of the antimicrobial compound, the selected nanostructure and the nature of the food or material where it is intended to be used; e.g., polymeric nanocapsules, which are formed by an oily core surrounded by a polymer layer, are suitable to load and deliver hydrophobic substances, and nanoliposomes, formed by a phospholipid bilayer with an aqueous core, can entrap and deliver both hydrophobic and hydrophilic molecules [27]. Recently, inorganic nanoparticles (NPs) have been demonstrated to have antibacterial activity against a wide range of microorganisms, including foodborne pathogens. Silver, copper, gold, titanium oxide and zinc oxide NPs, are associated with high efficacy at low concentrations and can be used to create antibacterial materials [28]. However, it is important to note that some nanostructures could present adverse biological effects to the consumer, and thus their safety must be assessed, evaluating important characteristics, such as concentration, dose, exposure route and duration, in addition to NP physiochemical properties, in order to assess their toxicity prior to use in the food industry [29,30].

### 3.1. Metal and Polymeric Nanoparticles

Several works have shown that some inorganic nanomaterials, such as metallic NPs, and nanostructured synthetic polymers are useful for preventing spoilage and pathogenic microbiota associated with dairy products like cheese and fluid milk when used as preservatives for direct mixture with the product or as nanocomposites in the packaging [31,32,33]. In this regard, nanocomposite films with better characteristics for food packaging can be produced from metal and other inorganic NPs, for example, titanium oxide, silver, zinc oxide, copper and gold. These elements could be incorporated with a film-forming solution and are associated with high efficacy at low concentrations, improving the antimicrobial, mechanical and barrier capabilities of the bioactive film [28]. In contrast with metal NPs, polymeric NPs are employed as a carrier and delivery system for antimicrobials through nanoencapsulation. The synthetic polymers most widely used in nanocomposites include polylactic acid (PLA), isotactic polypropylene and low-density polyethylene [29]. Nevertheless, recent works have shown the capability of biodegradable polymers such as poly-ε-caprolactone (PCL) and Eudragit to produce nanocapsules for the delivery of hydrophobic compounds (e.g., essential oils) and antimicrobial peptides in milk [34,35]. However, the formation of a protein corona around the polymeric NPs affects the delivery of antimicrobial peptides and represents a challenge for the use of these nanostructures in milk [35].

Other studies using silver nanoparticles (AgNPs) incorporated into biopolymers reported a reduction in microbial deterioration in dairy products. Incoronato and coworkers [36] used agar hydrosol with AgNPs to control *Pseudomonas* spp., coliforms and lactobacilli in Fior di Latte cheese stored at 10 °C. The authors reported that the silver-based packaging did not affect the functional dairy microorganisms; however, the system tested was able to inhibit the growth of spoilage bacteria. Ortega et al. [37] developed nanocomposite films based on corn starch and AgNPs able to extend the shelf life of fresh cheese samples for 21 days. The nanocomposite films inhibited the growth of *E. coli* and *Salmonella* spp., which are responsible for most foodborne diseases. Furthermore, these nanocomposites showed low toxicity to mammalian cells and improved cheese shelf life when the active package was developed by thermo-sealing the films [38]. In another study, the development of a polypropylene film coated with silica nanoparticles and *Pistacia atlantica* tree gum essential oil (GEO) was reported for milk packaging. The film containing silica nanoparticles and GEO presented a remarkable inhibitory effect (3.45 log CFU/g) against *S. aureus*, *S. enterica*, *E. coli* and *L. monocytogenes* and also extended the shelf life of milk by 35 days [39].

### 3.2. Lipid-Based Nanostructures

Lipid-based nanostructures have biodegradability, biocompatibility and a high diversity; these characteristics make lipid-based nanostructures very interesting for antimicrobial delivery in dairy products. The most studied lipid-based nanostructures include liposomes, solid lipid nanoparticles (SLN), nanostructured lipid carriers (NLCs) and nanoemulsions [24]. Each lipid nanostructure presents specific features according to the composition and production method. Liposomes are composed of amphiphilic lipids that can entrap both hydrophobic and hydrophilic molecules; nanoemulsions are dispersions in the configuration of oil-in-water (O/W) or water-in-oil (W/O) and, thus, are usually employed as a stabilizing structure for essential oils; SLNs that are produced from a single solid lipid species and NLCs formed from blends of liquid and solid lipids are used for lipophilic active compounds [40]. 

Probably the most studied lipid-based nanostructures for the encapsulation of antimicrobials for food applications, including dairy products, are liposomes due to their biocompatibility and low toxicity. The liposomal structure consists of self-assembled closed vesicles with one or more lipid bilayers surrounding an aqueous volume [41]. Diverse works have evaluated the use of liposomes as antimicrobial carriers for application in whole or skim milk, resulting in the control of *L. monocytogenes* using phosphatidylcholine (PC) liposomes loaded with bacteriocins [24,42]. Nisin has been the most studied antimicrobial for liposome encapsulation due to its compatibility and approval by regulatory agencies as a preservative for dairy foods. 

The encapsulation of the bacteriocin nisin in PC liposomes suggests that the strength of the association with lipid structure may depend on the phospholipid composition influencing the release from liposomes. The insertion of nisin into PC liposomes causes the stabilization of the nanovesicles, possibly through a lowering of curvature stresses [43]. Recently, Lopes et al. [44] reported that the presence of nisin promoted the formation of partial cubosome dispersion, through the transition of lamellar to cubic phase, presenting stability over a temperature increase. The results indicate that the complex network structure of the cubic phases can be efficient for a sustained release of nisin. In practical applications, these kinds of multilamellar vesicles provide a slow release of the entrapped or encapsulated content thanks to the specific structure, which can break layer after layer allowing a prolonged release of the content. Thereby, these characteristics can be interesting for applications requiring long-term storage and prolonged release, which are desirable properties for antimicrobials in dairy products.

### 3.3. Nanofibers

Nanofibers produced by electrospinning technology are an interesting alternative for food packaging and preservation due to their high yield, low production costs and high surface area (ranging from tens of nanometers to several micrometers). Nanofibers produced from synthetic and natural polymers can be used to fabricate a wide range of materials with unique features and properties such as light weight, easy processing and high loading capacity [45]. One of the most important applications of electrospun nanofibers is the development of innovative food packaging because antimicrobials, antioxidants and other bioactive compounds can be easily integrated into nanofibers during electrospinning [46].

Compared to other encapsulation methods, an advantage of electrospinning is the absence of heat, due to the bioactives being entrapped by fibers by combining them with the polymer solution prior to the electrospinning process which occurs at room temperature, which is important for maintaining the efficacy of encapsulated thermolabile compounds, such as essential oils, during nanofiber production [47]. In relation to their use in the dairy industry, researchers are developing antimicrobial and active food packaging materials to extend the shelf life and can also be used as sensors for determining changes in pH or temperature during storage. Liu et al. [48] produced an intelligent starch/poly-vinyl alcohol film with the addition of anthocyanin and limonene which was capable of monitoring pH changes and showed inhibiting activity against *B. subtilis*, *S. aureus* and *Aspergillus niger* in pasteurized milk. In another research study, an antimicrobial packaging material was developed using allyl isothiocyanate (AIC) in the vapor phase. The adhesive composite produced with gelatin electrospun fibers containing AIC (2%, *v*/*v*) showed antimicrobial activities against *S. aureus* ATCC 25,923 and Shiga-toxin-producing *E. coli* O157:H7. In addition, gelatin nanofibers containing AIC (10%, *v*/*v*) extended the shelf life of cheese from 4 weeks to 8 weeks [49].

### 3.4. Nanofilms and Nanocoatings

In the food industry, packaging films, edible films and coatings are considered a sustainable and biodegradable alternative that presents many advantages when compared to conventional packaging; for example, they reduce waste, contribute to packaging material efficiency, prolong shelf life and preserve food quality [50]. Due to their versatility of production, the wide range of materials that can be used and the ability to carry various active compounds, including antioxidants and/or antibacterial agents, active films, edible films and coatings are one of the most promising areas in food science [51,52].

Nowadays, one of the challenges with the utilization of nanotechnology in films and coatings is related to their high water vapor permeability and poor mechanical properties as compared with synthetic materials [53]. However, the use of nano-scale structures provides some advantages to the films and coatings such as the high stability on the food matrix surface, facility of preparation and lower concentration of materials required [52]. Most of the research on nanostructured films and coatings for the dairy industry has been focused on the control of spoilage and pathogenic microorganisms to ensure better quality and safety of cheeses. In this regard, Leite et al. [54] reported the fabrication of gelatin films with the addition of rosin-grafted cellulose nanocrystals (r-CNCs) for antimicrobial packaging applications in mozzarella cheese. The developed films presented antimicrobial activity against *E. coli* and *S. aureus* (MIC 22 mg/mL and MIC 5.5 mg/mL, respectively), and r-CNC gelatin films presented inhibitory activity on agar plates and extended the shelf life of cheese samples. The development of antibacterial films based on alginate, spherical AgNPs and lemongrass essential oil was also reported. This smart film exhibited strong antibacterial activity against *S. aureus*, *E. coli*, *Bacillus cereus* and *Salmonella* Typhi, preserving the color, surface texture and softness of cheese for 14 days. In addition, the film changed its color (darkened) as a function of temperature and light exposure [55].

## 4. Nanostructured Antimicrobials in Dairy Industry

The application of nanostructured antimicrobials in milk and dairy products has been investigated. Nanotechnology approaches can be useful to improve the quality and safety of dairy products, through the delivery and controlled release of antimicrobial additives and the development of active food packaging. 

In the next sections, some relevant examples of the use of nanostructured antimicrobials in milk and dairy products are presented and discussed. Studies including the evaluation and effectiveness of antimicrobial nanostructures on dairy products and packaging materials intended for the dairy industry are summarized in Table 1.

### 4.1. Milk as a Model System

The use of additives in fluid milk is often forbidden by the legislation of most countries; nevertheless, this food has been used as a model in many studies. This fact is due to milk’s unique chemical composition and, therefore, makes it an interesting substrate to study the effectiveness of nanomaterials. Because interaction with fat and proteins can hamper the application of some antimicrobials in real food systems, the testing on milk as a model system can provide useful information during the formulation of antimicrobial nanostructures.

Milk has been mainly used as a model food to investigate the efficacy of antimicrobial nanoliposomes. Most studies showed that the effectiveness is improved in skim milk when compared to whole milk, reinforcing the idea that the interaction of some antimicrobial molecules with fat can be a relevant factor for the reduction in antimicrobial activity in real food matrices [22,71]. Phosphatidylcholine (PC) liposomes containing the bacteriocin nisin were produced by the thin film method, showing a mean particle size of 140 nm, reducing the counts of *L. monocytogenes* from 4.5 log CFU/mL to below the detection limit of the method in both whole and skim milk during 14 days of refrigeration storage at 7 °C [56]. The same system was used for the encapsulation of the antimicrobial peptide P34, but in this case, the control of *L. monocytogenes* was only achieved in skim milk for up to 8 days at 7 °C [57]. The antimicrobial effect of liposome-encapsulated nisin was also observed against *L. monocytogenes* Scott A in fluid milk by a significant increase in the lag phase after incubation at either 5 °C or 20 °C [72]. In another study, liposomes prepared with PC and 1,2-dioleoyloxy-3-trimethylammonium-propane were used for the encapsulation of sakacin (*L. sakei* bacteriocins). These liposomes controlled the growth of *L. monocytogenes* in goat milk, and an about 5 log difference was observed in comparison with control for up to 5 days at 7 °C [58]. The effective antimicrobial activity of encapsulated bacteriocins in milk for at least 5 days under refrigeration is relevant as this is the period in which the product should be consumed after the milk package is opened.

Polymeric nanocapsules have been tested as an interesting vehicle for the delivery of lipophilic antimicrobials, such as essential oils. The encapsulation of the essential oil from *Baccharis dracunculifolia* in Eudragit was achieved by the nanoprecipitation method, resulting in nanoparticles of about 150 nm and 99% encapsulation efficiency. When the antimicrobial activity against *L. monocytogenes* was tested in milk, the inhibitory effect was missing in whole milk, while a significant delay in the lag phase was observed in skim milk [34]. The peptide P34 was also encapsulated into poly-ε-caprolactone and Eudragit nanocapsules, and although these nanostructures were inhibitory to *L. monocytogenes* in agar plates, antimicrobial activity was not observed in skim or whole milk, possibly due to the protein corona effect caused by milk casein [35].

Garlic extract was encapsulated into nanoliposomes and tested against different strains of *Listeria* spp. in milk at a temperature abuse of 37 °C. This formulation caused a 4 log reduction in the viable cell counts of 80% of *Listeria* strains tested after 10 h incubation [59]. Moreover, considering that the co-encapsulation of different antimicrobials may improve the effectiveness and broaden the inhibitory spectrum against food pathogens, garlic extract was also co-encapsulated with nisin [60]. The growth of food pathogens like *L. monocytogenes*, *S. aureus*, *Salmonella* Enteritidis and *E. coli* in whole milk at 37 °C resulted in a difference of 1–4 log CFU/mL by exposure to liposome-encapsulated nisin/garlic when compared with free nisin and garlic extract separately and 3–6 log CFU/mL when compared to the control. When the effect of co-encapsulation was tested on *L. monocytogenes* under refrigeration (7 °C) for up to 25 days, viable counts were 4–5 log CFU/mL lower than the control values.

**Figure 2:**
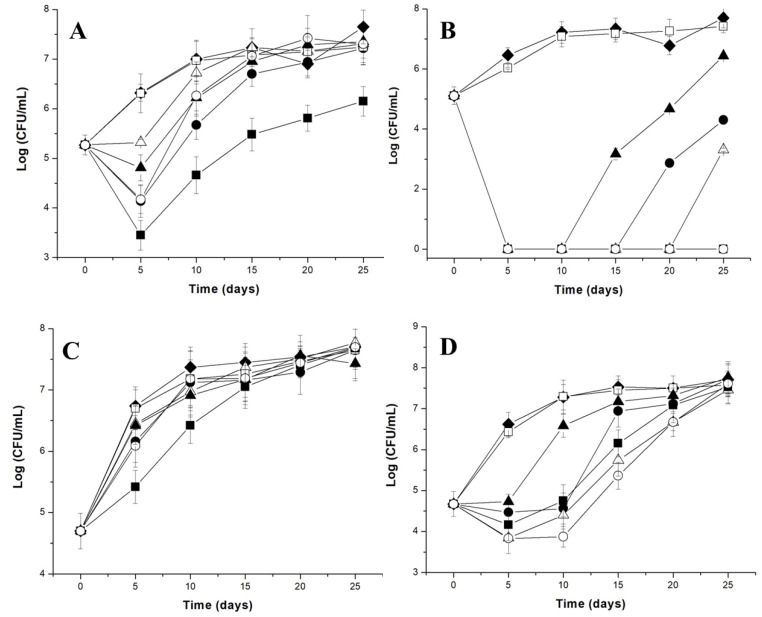
Growth curves of *L. monocytogenes* in whole milk (**A**) or skim milk (**B**) and a cocktail of *Listeria* strains in whole milk (**C**) or skim milk (**D**) during incubation at 7 °C. The cocktail of *Listeria* strains contained a mixture of *L. monocytogenes* 4b, *L. innocua* 6a, *Listeria* sp. Str1 and *Listeria* sp. Str2. Viable cell counts of the control (filled rhombus), PC liposomes (filled circle), PC–pectin (filled square), PC–polygalacturonic acid (filled triangle), free lysozyme (open square), free nisin (open triangle) and free lysozyme–nisin (open circle) were evaluated. (Reproduced from [75] with permission).

Nanoliposomes with sizes ranging from 94 to 160 nm encapsulating the bacteriocin nisin were prepared with PC coated with polysaccharides (pectin or polygalacturonic acid). The inhibitory activity of these nanostructures was recorded against five different strains of *Listeria* in milk–agar plates, with a better efficacy against *Listeria innocua* strain 6a [73]. Effective control of Gram-positive bacteria by combinations of nisin and lysozyme has been reported in food systems, in addition to inhibiting *S.* Typhimurium and *E. coli* [74]. Lysozyme may help the contact of nisin with the cell membrane, resulting in an increased bacterial death rate, but the actual mechanism of synergy between nisin and lysozyme is not clearly understood. Based on this premise, liposomes co-encapsulating nisin and lysozyme were tested in fluid milk as a model food. During the tests conducted at a temperature abuse of 37 °C, PC–pectin liposomes reduced the viable counts of *L. monocytogenes* by 2 log CFU/mL and 5 log CFU/mL in whole and skim milk, respectively [75]. As shown in Figure 2, under refrigeration temperature (7 °C), the population of *L. monocytogenes* was reduced to below the detection limit for up to 25 days in skim milk (Figure 2B). The results from this study suggest that nanoliposomes containing polysaccharides, such as pectin and polygalacturonic acid, can be a valuable system for the controlled delivery of nisin and lysozyme in dairy foods.

### 4.2. Antimicrobial Nanostructures as Additives in Dairy Products

A great diversity of dairy products is currently marketable, including a broad variety of cheeses, butter, yogurt, cream, ice cream, pudding/flans and fermented milks. Among these, white soft cheese is particularly susceptible to becoming contaminated with pathogenic and spoilage microorganisms during storage. As described above for fluid milk, natural antimicrobials can be applied as biopreservatives, but their antimicrobial effectivity can be reduced by undesirable interactions with constituents of the food matrix. In this regard, encapsulation may overcome some problems associated with the incorporation of free antimicrobials in dairy foods, including cheeses. Among encapsulation methodologies used in food systems, nanoliposomes are described as one of the most interesting methods for the encapsulation of natural antimicrobials, such as essential oils, bacteriocins and other antimicrobials [10,42,76]. Some examples of nanoliposome application in cheese production with the aim of increasing shelf life are presented in the sequence.

The antimicrobial peptides nisin and P34 were encapsulated in partially purified soybean PC and PC–cholesterol (7:3) liposomes and tested in Minas frescal, a typical Brazilian soft cheese. A significant reduction in the viable counts of *L. monocytogenes* was observed for all treatments as compared to the control during 21 days of storage at 7 °C. However, encapsulation in PC–cholesterol liposomes was less efficient in controlling *L. monocytogenes* growth in comparison with free and PC liposome-encapsulated bacteriocins. The maximum inhibitory effect was observed for nisin and P34 encapsulated in PC liposomes after 10 days of cheese storage [77]. The PC liposomes containing nisin were also compared with free nisin to control *L. monocytogenes* inoculated on the surface of cheese samples. A bacteriostatic effect was observed for encapsulated nisin while free nisin showed a bactericidal effect [69]. Differences in the effect of free and liposomal nisin may indicate that nisin has been strongly associated with the phospholipid, being gradually released from liposomes, as previously suggested for fluid milk [78]. 

The effect of cumin essential oil (CEO) nanoemulsion on the quality of white soft cheese was reported [79]. This nanoemulsion showed broad antimicrobial activity against different pathogens like *S. aureus*, *B. cereus*, *L. monocytogenes*, *E. coli*, *S.* Typhimurium, *Pseudomonas aeruginosa*, *Yersinia enterocotilica*, *A. niger* and *Aspergillus flavus*. CEO nanoemulsions were used as a preservative solution for the white soft cheese in different ratios (0.50, 0.75 and 1.00%). The results showed that yeasts and molds and psychrotrophic counts were not detected for cheese preserved in solutions containing 1% nanoemulsion during the storage period of 60 days. However, for cheese preserved in 0.50 and 0.75% of nanoemulsion solutions, minor counts were observed. Concerning the organoleptic properties, it was found that the highest total scores were given to cheeses preserved in the same concentrations (0.50 and 0.75%) of CEO nanoemulsion solutions. From these results, the study recommended using CEO nanoemulsion to preserve the quality of white soft cheese, being used as a natural preservative during storage [79]. Thus, the encapsulation of antimicrobial compounds into lipid nanostructures represents a promising alternative to control foodborne pathogens in cheeses.

Dairy products such as yogurt and fermented milks are often less susceptible to microbial degradation due to the predominance of starter cultures and the accumulation of lactic acid during fermentation. However, examples of contamination with spoilage and pathogenic microorganisms have been related to the utilization of poor-quality raw milk and failures in good hygienic practices during the manufacturing process and storage [2,80]. Electrospun nanofibers of sodium alginate were used for the encapsulation of probiotic *L. brevis* cells and incorporated into a functional yogurt drink. The encapsulated probiotic showed an important increase in the survival ratio as compared with free bacteria, suggesting that nanofiber encapsulation has the potential to increase the benefits of a functional beverage [81]. Considering that lactic acid bacteria are largely associated with the production of antimicrobial substances in dairy products, the maintenance of *L. brevis* viability should be beneficial for product safety as well.

### 4.3. Antimicrobial Nanostructures in Packaging Applications

The development of packaging materials with improved properties to warrant food safety and quality is a topic of utmost relevance in the dairy industry. Therefore, a variety of nanocomposite active packaging with potential application in dairy products has been described [82].

Active films prepared with either petroleum-based or natural polymers have been intensively investigated, and several formulations are reported to control foodborne pathogens in vitro and in situ [52]. The effectiveness of antimicrobial packaging formulations has been proven on milk agar as a model system and also on real dairy foods. Antimicrobial films of conventional plastics (e.g., polyethylene, polypropylene) incorporating metallic nanoparticles such as silver (Ag) and zinc oxide (ZnO) have been described as effective to control microbial growth in cheeses [83,84,85]. However, increased attention has been devoted to the use of biopolymers for the development of edible and/or biodegradable food packaging for dairy applications as well [82]. 

As an example of using biopolymers, casein and gelatin nanocomposite films containing liposome-encapsulated nisin and halloysite nanoclay were characterized as an interesting alternative for active food packaging. These films showed antimicrobial activity against *L. monocytogenes*, *B. cereus* and *Clostridium perfringens* when tested in agar plates prepared with skim milk [86]. Moreover, casein films showed better physical properties as compared with gelatin films, and they were less rigid and very elastic, compatible with dairy applications. Antimicrobial nanocomposite films prepared with polypropylene, nisin and montmorillonite nanoclay were also produced and tested as potential food packaging. When tested on milk agar plates, the nanocomposite films inhibited *L. monocytogenes*, *S. aureus* and *C. perfringens*, and the antimicrobial activity was released for up to 48 h during incubation in simulant solutions of fat and acid foods [87].

A nanocomposite antimicrobial packaging was developed with corn starch as a polymeric matrix, using nisin or pediocin as natural preservatives and halloysite nanoclay as a filler to promote film reinforcement. In these formulations, an innovative approach was tested by incorporating the bacteriocins adsorbed on halloysite before incorporation into film-forming solutions. Antimicrobial films were obtained showing the inhibition of *L. monocytogenes* and *C. perfringens* in milk agar, although the addition of nanofiller retained the antimicrobial activity as compared with films without nanoclay addition [88]. The nanocomposite film formulation containing nisin was also produced by the direct melt-extrusion method in addition to control films without nisin and films containing starch and glycerol only. The antimicrobial activity was tested in skim milk agar against *L. monocytogenes*, *S. aureus* and *C. perfringens*, which were all inhibited by nisin-containing nanocomposites. These films were tested as active packaging for soft cheese previously inoculated with *L. monocytogenes*. The bacterial counts were significantly reduced by antimicrobial films with 2 g/100 g nisin while those containing 6 g/100 g nisin completely inhibited *L. monocytogenes* after 4 days [89]. Thus, starch/halloysite/nisin nanocomposite films can be a valuable barrier to control microbial contamination in cheese.

**Figure 3:**
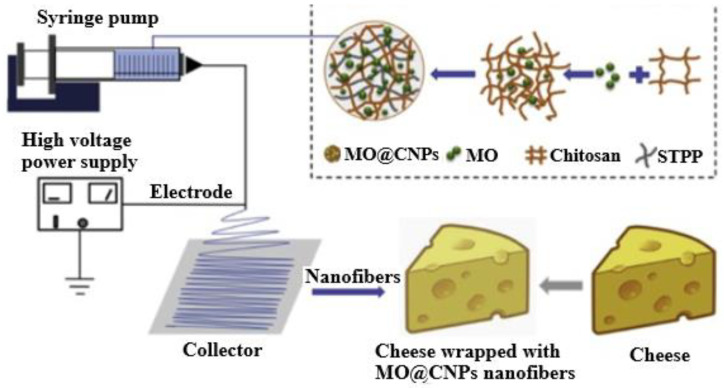
Schematic representation of electrospinning for moringa-oil-loaded chitosan nanoparticle (MO@CNP) nanofibers. (Reproduced from [64] with permission).

Two cheese varieties, a rennet-curd (gouda) and an acid-curd (quark) cheese, were tested with the addition of furcellaran nanocomposite film with silver nanoparticles by Pluta-Kubica et al. [90]. The cheese quality was examined, indicating that the use of the film improved the microbiological quality of cheeses during storage, slowing down and inhibiting the growth of yeast in gouda and quark, respectively. In addition, regarding gouda, an inhibitory effect of this film on mold count was also observed. In another study, Lin and collaborators [64] developed a food packaging against *L. monocytogenes* and *S. aureus* on cheese (Figure 3). For this, fabricated moringa-oil-loaded chitosan nanoparticles (MO@CNPs) were embedded in gelatin nanofibers by the ionic crosslinking method. The sensory quality of cheese was not affected by the packaging, and MO@CNP nanofibers showed high antibacterial activity when applied on the cheese at 4 °C and 25 °C for 10 days.

Nanofibers of the biodegradable polymer poly(butylene adipate-co-terephthalate) prepared by the electrospinning technique were used as carriers for the antimicrobial peptide nisin. PBAT fibers were fully characterized, and nisin was well dispersed throughout the nanofiber. These antimicrobial fibers inhibited *L. monocytogenes* in milk agar [91]. The study provided insights about the preparation of nisin–PBAT nanofibers by the electrospinning technique, indicating their application in the food packaging industry. Moreover, electrospun poly(ε-caprolactone) nanofibers containing the natural antifungal natamycin were produced as a potential packaging material. These nanofibers showed large inhibition zones against different fungal strains cultivated in skim milk agar as a food model [70]. A gradual migration of natamycin from the polymeric fibers was observed in food simulating solutions. The antifungal activity of natamycin-containing nanofibers was also established in samples of soft cheese as a real food system. A clear growth inhibition of toxigenic strains of *A. flavus* and *Penicillium citrinum* was observed at the cheese/nanofiber mat interface.

## 5. Toxicity of Nanostructured Antimicrobials

Nanostructured materials present different physical, chemical and biological properties as compared with their respective bulk counterparts. Considering that nanomaterials are complex systems, adequate knowledge of their characteristics such as size, shape, surface area, surface charges, composition, purity, dispersion and solubility is quite important to understand how they interact with biological environments [30]. The potential toxicity of a nanoparticle depends on complex physicochemical properties, which influence its stability and should be evaluated in vitro and in vivo [92]. It is therefore worthwhile to improve our understanding of the bioactivity and toxicity aspects of foods that have been exposed either directly or indirectly to nanostructured antimicrobials [29,93].

Antimicrobial nanomaterials used in food are probably ingested as the main route of direct entry into higher organisms. After oral exposure, the adsorption, distribution, metabolism and excretion (ADME) can be different as compared with the same material in bulk state, and nanostructured antimicrobials are possibly absorbed in the gastrointestinal tract into the circulatory system by modified pathways [30,93]. Nanocomposite active packaging is a major topic in dairy nanotechnology. Nanostructured antimicrobials can be valuable to promote a controlled diffusion, improve cost-effectiveness and increase shelf life, but limited data about the migration of nanoparticles to the food matrix and their potential toxicity have attracted significant concern [94,95]. Besides the usual concerns on metallic NPs, delivery systems based on nanostructured lipids have gained attention as encapsulated lipophilic substances apparently change the general absorption pathway and may cause unknown effects [96,97].

Limited research has been specifically conducted on the toxicity of nanostructured antimicrobials in dairy products. The cytotoxicity of nanocomposite films containing AgNPs used as antimicrobial cheese packaging was tested in different mammalian cells. Although the films showed low cytotoxicity for Caco-2 cells, some difficulties were observed for Vero cell adhesion and differential marker expression in monocytes and macrophage THP-1 cells [38]. The migration of AgNPs from breast milk storage bags into milk was investigated under different conditions. Commercially available breast milk storage bags labeled with the presence of nanosilver were investigated, including four LDPE bags and five multilayer PET/PE bags. The study showed the absence of Ag migration from breast milk storage bags into milk under various simulated migration conditions [98]. A nanocomposite antibacterial prepared from chitosan nanoparticles and the bacteriocin microcin J25 was tested against tetracycline-resistant enterotoxigenic *E. coli* (ETEC) in milk and other food simulants. The nanostructured antimicrobial caused a significant reduction in bacterial counts after 2 h incubation in skim milk [99]. The antimicrobial caused no cytotoxic effect on mouse RAW264.7 cells, while LPS-induced toxicity was significantly reduced, and inflammatory response was significantly ameliorated. 

## 6. Conclusions and Perspectives

Owing to their remarkable properties, both organic and inorganic nanostructured antimicrobials have engendered several interesting fields in dairy science and technology. Considering the current scenario in which the dairy industry faces many challenges, especially the need to attend to consumers who are looking for natural and minimally processed food, the incessant investigation for the application of nanostructures has led to the development of practical productions and the commercialization of products in some cases. However, realizing the application of these nanostructured materials at a large scale in the economic setup is still a huge challenge. Therefore, designing novel, applicable and inexpensive methodologies for the scaled-up manufacturing of these materials should be improved in order to increase access to high-quality and innovative products. The use of byproducts from the food industry as low-cost and biocompatible encapsulating materials for antimicrobial delivery merits additional investigation. Moreover, further studies should be conducted on the potential toxicity and stability of nanostructured antimicrobials in real dairy products. Such achievements could bring substantial improvements in dairy food for future generations.

## Figures and Tables

**Table 1:** Antimicrobial nanostructures for control of microbial pathogens in milk and dairy.

Dairy Product	Nanostructure *	Antimicrobial	Target Bacteria	Main Results	Ref.
Fluid milk	PC liposomes	Nisin	*L. monocytogenes*	Reduction in viable counts in whole and skim milk	[56]
	PC liposomes	Peptide P34	*L. monocytogenes*	5 log reduction in skim milk	[57]
	PCL and Eudragit nanocapsules	Peptide P34	*L. monocytogenes*	Protein corona hinders antimicrobial activity in milk	[35]
	PC/DOTAP liposomes	Sakacin	*L. monocytogenes*	5 log reduction in goat milk	[58]
	Eudragit RS100 nanoparticles	*Baccharis dracunculifolia* EO	*S. aureus*, *B. cereus*, *L. monocytogenes* and *S.* Enteritidis	2 log reduction in skim milk	[34]
	PC liposomes	Garlic extract	*L. monocytogenes*	4 log reduction in whole milk	[59]
	PC liposomes	Nisin–garlic extract	*L. monocytogenes*, *S. aureus*, *E. coli*, *S*. Enteritidis	Synergic effect in the control of pathogens in whole milk	[60]
	Metallic nanoparticles	Magnesium oxide nanoparticles in combination with nisin	*E. coli* and *S. aureus*	Synergic effect in the control of the pathogens in milk	[61]
Cheese	Thymol-loaded nanofiber	Thymol	*Aspergillus parasiticus*	Prevented the growth of *A. parasiticus* on Kashar cheese	[62]
	Amaranth protein isolate: pullulan nanofibers	Nisin	*Sakmonella* Typhimurium, *L. monocytogenes* and *L. mesenteroides*	Inactivation of *S.* Typhimurium, *L*. *monocytogenes* and *L*. *mesenteroides* in fresh cheese	[63]
	Moringa oil/chitosan nanoparticles embedded gelatin nanofibers	Moringa-oil-loaded and chitosan	*L. monocytogenes* and *S. aureus*	High antibacterial activity at 4 °C and 25 °C for 10 days, without any effect on the sensory quality of cheese	[64]
	Layer-by-layer electrostatic self-assembled coatings based on flaxseed gum and chitosan	Chitosan	*E. coli* and *S. aureus*	Inhibition of molds, *yeasts, E. coli* and *S. aureus* in Mongolian cheese	[65]
	PLA films containing zero-valent iron (ZVI)	Fe_2_O_3_ nanoparticles	Spoilage bacteria and fungi	Inhibited the growth of spoiled microorganisms in goat cheese	[66]
	LDPE films	Ag, CuO and ZnO nanoparticles	Coliforms	Decrease 4.21 log CFU/g of coliforms in ultra-filtrated cheese after 4 weeks of storage at 4 ± 0.5 °C	[67]
	EOs-zein nanofibers	EOs of *Laurus nobilis* and *Rosmarinus officinalis*	*L. monocytogenes* and *S. aureus*	Reduction of ~2 log units of *L. monocytogenes* and *S. aureus* in cheese slices	[68]
	Safflower oil nanoemulsion	Curcumin	*S. aureus* and *E. coli*	Improved sensory evaluation of cheese. Antibacterial activity against *S. aureus* and *E. coli*	[69]
Milk agar (model system)	PCL nanofibers	Natamycin	Filamentous fungi and yeasts	Inhibition zones ranged from 4.3 to 25.6 mm of fungi in skim milk agar	[70]

* PC, phosphatidylcholine; PCL, poly-ε-caprolactone; DOTAP, 1,2-dioleoyloxy-3-trimethylammonium-propane; PLA, poly-L-lactic acid; LDPE, low-density polyethylene; EO, essential oil.

## Data Availability

No new data were created in this study. Data sharing is not applicable to this article.
